# A Pre-clinical Animal Model of Secondary Head and Neck Lymphedema

**DOI:** 10.1038/s41598-019-54201-2

**Published:** 2019-12-04

**Authors:** Giulia Daneshgaran, Andrea Y. Lo, Connie B. Paik, Michael N. Cooper, Cynthia Sung, Wan Jiao, Sun Y. Park, Pauline Ni, Roy P. Yu, Ivetta Vorobyova, Tea Jashashvili, Young-Kwon Hong, Gene H. Kim, Peter S. Conti, Yang Chai, Alex K. Wong

**Affiliations:** 1 Albert Einstein College of Medicine, 1300 Morris Park, Bronx, NY 10461 USA; 2 Division of Plastic and Reconstructive Surgery, Keck School of Medicine of USC, 1510 San Pablo St. Suite 415, Los Angeles, CA 90033 USA; 3 Tulane University School of Medicine, 1430 Tulane Ave, New Orleans, LA 70112 USA; 4 Department of Molecular and Cell Biology, University of California Berkeley, 2121 Berkeley Way, Berkeley, CA 94720 USA; 5 Molecular Imaging Center, Keck School of Medicine of USC, 2250 Alcazar St, Los Angeles, CA 90089 USA; 6 Department of Surgery, Keck School of Medicine of USC, 1975 Zonal Ave., Los Angeles, CA 90033 USA; 7 Department of Dermatology, Keck School of Medicine of USC, 1975 Zonal Ave., Los Angeles, CA 90033 USA; 8 Center for Craniofacial and Molecular Biology, Herman Ostrow School of Dentistry of USC, 2250 Alcazar St, Los Angeles, CA 90089 USA

**Keywords:** Animal disease models, Head and neck cancer

## Abstract

Head and neck lymphedema (HNL) is a disfiguring disease affecting over 90% of patients treated for head and neck cancer. Animal models of lymphedema are used to test pharmacologic and microsurgical therapies; however, no animal model for HNL is described in the literature to date. In this study we describe the first reproducible rat model for HNL. Animals were subjected to two surgical protocols: (1) lymphadenectomy plus irradiation; and (2) sham surgery and no irradiation. Head and neck expansion was measured on post-operative days 15, 30 and 60. Magnetic resonance imaging (MRI) was acquired at the same time points. Lymphatic drainage was measured at day 60 via indocyanine green (ICG) lymphography, after which animals were sacrificed for histological analysis. Postsurgical lymphedema was observed 100% of the time. Compared to sham-operated animals, lymphadenectomy animals experienced significantly more head and neck swelling at all timepoints (P < 0.01). Lymphadenectomy animals had significantly slower lymphatic drainage for 6 days post-ICG injection (P < 0.05). Histological analysis of lymphadenectomy animals revealed 83% greater subcutis thickness (P = 0.008), 22% greater collagen deposition (P = 0.001), 110% greater TGFβ1^+^ cell density (P = 0^.^04), 1.7-fold increase in TGFβ1 mRNA expression (P = 0.03), and 114% greater T-cell infiltration (P = 0.005) compared to sham-operated animals. In conclusion, animals subjected to complete lymph node dissection and irradiation developed changes consistent with human clinical postsurgical HNL. This was evidenced by significant increase in all head and neck measurements, slower lymphatic drainage, subcutaneous tissue expansion, increased fibrosis, and increased inflammation compared to sham-operated animals.

## Introduction

Lymphedema is a debilitating condition caused by insufficiency of the lymphatic system that affects over 250 million individuals worldwide^1^. It is characterized by progressive soft tissue swelling and can carry a significant disease burden^2^. Primary lymphedema due to inborn defects of lymphangiogenesis is rare, with an estimated incidence of 1:6,000–10,000 live births^3^. In the US, secondary lymphedema is far more common and is mainly caused by lymphatic injury from surgery, radiation, trauma, infection, or obesity^4,5^. In particular, cancer patients who have received surgical and/or radiation treatments are at high risk for developing lymphedema^6^. Specifically, secondary head and neck lymphedema (HNL) is frequently seen in patients treated for head and neck cancer, which affects over 550,000 people annually. Of these head and neck cancer patients, HNL can be seen in up to 90% of cases, although it is frequently misdiagnosed by generalist physicians as other conditions causing head and neck swelling^7–9^.

Lymphatic disruption in HNL results in chronic swelling of external and/or internal soft tissue structures of the head and neck, leading to visibly abnormal swelling of the cheeks, eyelids, and other facial structures. Such intense swelling can ultimately interfere with a patient’s quality of life, impairing vision, hearing, speech, swallowing, eating, and breathing, with the risk of airway compromise and respiratory obstruction in severe cases^10–12^. Stasis of lymph fluid leads to inflammatory changes which impact skin barrier function and put patients at risk for life-threatening cellulitis^13^. Chronic facial pain can also cause significant psychological distress. Lastly, HNL is a disfiguring stigma of cancer that bears with it an immeasurable psychological and emotional burden^14^.

HNL is underappreciated by clinicians due to limited awareness of the disease and its variability in clinical manifestations, often leaving affected patients without a definitive diagnosis. In addition, no definitive cure for HNL has been established in peer-reviewed literature. At present, different management modalities for HNL have been attempted to reduce symptoms^10,15,16^. However, these modalities are difficult to execute for many patients and are associated with poor long-term adherence given their time-consuming and costly nature^17^. For many patients, more extensive surgical therapy becomes the only management option^18–20^. The current management of HNL is not standardized across institutions and has not been studied with long-term patient-reported outcomes. In addition, given the challenges of performing compression bandaging and manual lymphatic drainage on structures in the head and neck, standard treatment for other forms of lymphedema cannot be reasonably and reproducibly used to manage HNL.

Overall, the lack of fundamental understanding of HNL on the part of clinicians and scientists has had a negative impact on the development of effective treatment modalities. The greatest opportunity to impact this disease lies in early identification of patients at risk followed by implementation of strategies to prevent pathophysiologic progression. To better understand the pathophysiology of HNL and cultivate a platform for testing potential therapeutic interventions, it is necessary to have a faithful and easily reproducible animal model of HNL. Animal models can be used to test the entire spectrum of current treatment modalities as well as potential pharmacological therapeutics specific to HNL. Careful review of the published literature reveals that no animal model for HNL has been described to date in the peer-reviewed scientific literature^21,22^. Thus, our primary objective was to develop the first reproducible animal model for HNL that can be used by future researchers to test therapies specific to this underdiagnosed and poorly managed disease.

## Methods

### Animals

As previously described in our published work on animal models of lymphedema, all experiments were performed in accordance with the statutes from *The Guide for the Care and Use of Laboratory Animals* (National Research Council, 8th ed, 2011), the Animal Welfare Act, and other Federal regulations^23^. Our experimental protocol was approved by the University of Southern California Institutional Animal Care and Use Committee. Prox1-enhanced green fluorescent protein (Prox1-EGFP) transgenic rats, 12–16 weeks old and weighing 200–300 g were bred within the University of Southern California animal facility. All animals were housed in a light- and temperature-controlled environment with access to food and water *ad libitum*.

### Experimental design

**Figure 1:**
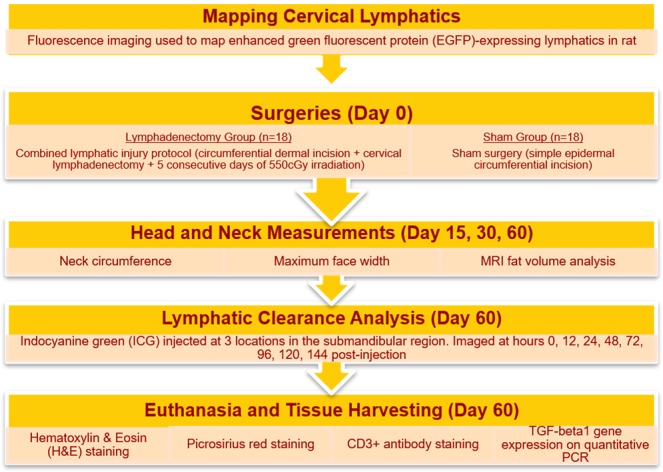
Timeline of the experimental methodology. Before surgery, cervical lymph nodes were mapped using Prox1-EGFP expressing lymphatic reporter rats. On the day of surgery, animals were randomized to the lymphadenectomy group or the sham surgery group. Head and neck measurements and MRI scans were acquired pre-operatively and then post-operatively on days 15, 30 and 60. Lymphatic clearance analysis using ICG lymphography and tissue harvesting were conducted at the final time point.

The design and timing of our experimental setup is described in Fig. 1. Fourty (40) Prox1-EGFP expressing lymphatic endothelial cell reporter rat were separated into two groups: (1) lymphadenectomy animals receiving the combined lymphatic injury protocol and (2) control animals receiving sham surgery. Based on a power calculation and accounting for 20% mortality rate based on prior experience, N = 10 animals were required per group to achieve statistical significance^24^. To ensure success, we elected to double the number of animals, thus yielding N = 20 animals per group. Sham surgery consisted of a scratch incision that did not disrupt the epidermal surface while the animal was maintained under isoflurane-inhaled anesthesia. Control animals did not undergo radiation therapy. Using our previous experience with animal models of lymphedema, we decided to exclude from analysis all animals that developed tissue necrosis or surgical site infection were sacrificed and in order to prevent confounding of experimental results (in total, 4 out of 40 animals met exclusion criteria: 2 from the lymphadenectomy group and 2 from the control group)^23^.

### Anatomical mapping of cervical lymphatics

**Figure 2:**
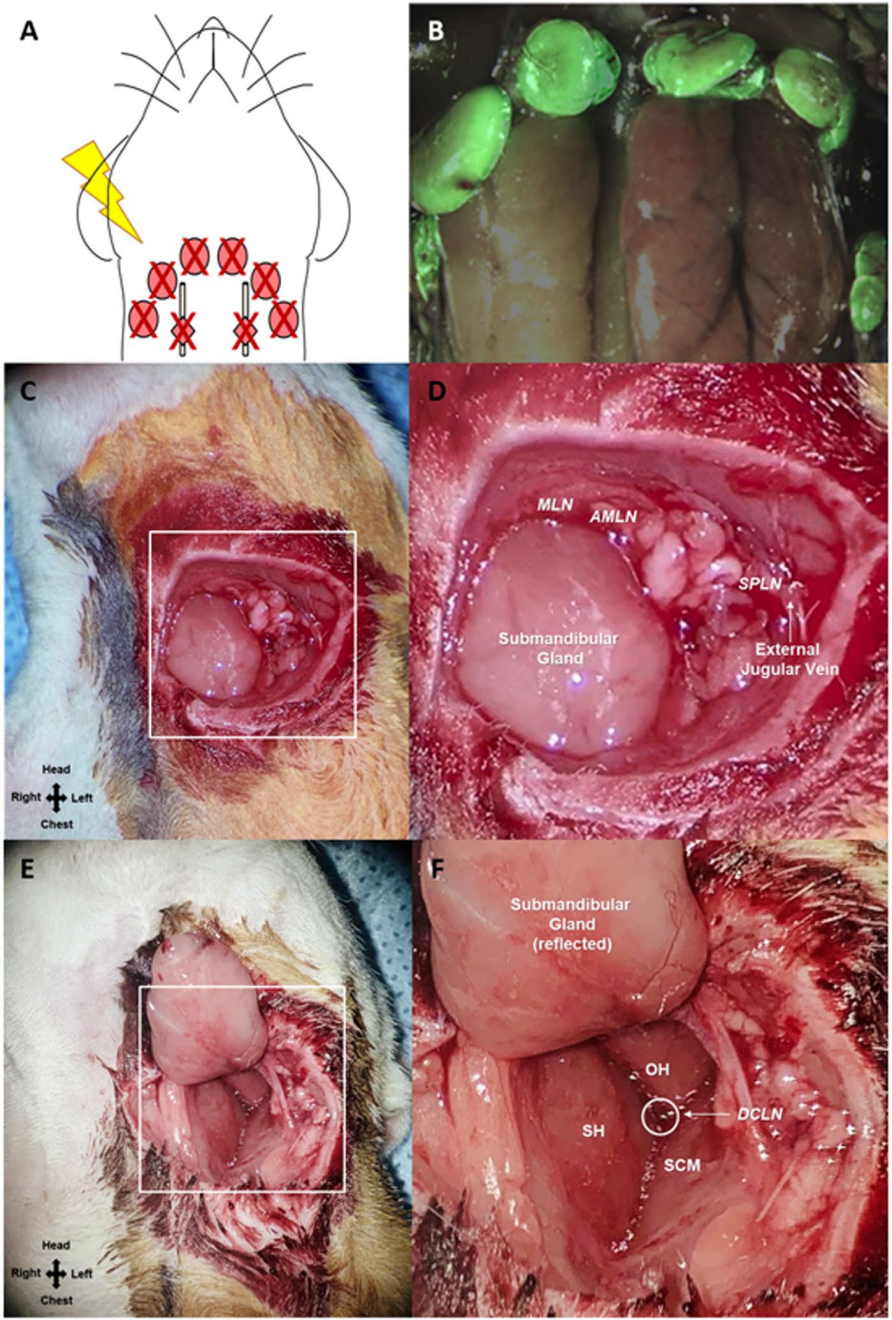
Schematic of the cervical lymphadenectomy model, including identification and removal of cervical lymph nodes using Prox1-EGFP lymphatic reporter rats. (**A**) To induce cervical lymphedema, rats underwent surgical dissection of the superficial cervical lymph nodes (red ovals) and deep cervical lymph nodes (red diamonds overlying the carotid sheath) followed by irradiation. (**B**) Superimposed images of superficial cervical lymph nodes located around the submandibular glands and the same lymph nodes under green fluorescence microscopy in a Prox1-EGFP lymphatic reporter rat. (**C**) Intraoperative image of rat neck identifying the field of view for 2D. (**D**) Identification of the superficial cervical lymph nodes: mandibular lymph node (MLN) located superiomedial to the submandibular gland, accessory mandibular lymph node (AMLN) located superiolateral to the submandibular gland, superficial parotid lymph node (SPLD) overlying the external jugular vein. (**E**) Intraoperative image of rat neck identifying the field of view for 2F. (**F**) Identification of the deep cervical lymph nodes (DCLN) located deep to the junction of the sternohyoid (SH), omohyoid (OH) and sternocleidomastoid (SCM) muscles and overlying the carotid sheath.

We have previously developed and described a bacterial artificial chromosome (BAC)-based lymphatic reporter rat, in which enhanced green fluorescent protein (EGFP) is expressed under the regulation of the Prox1 promoter^25^. This EGFP-expressing lymphatic reporter rat permits us to conveniently visualize under fluorescence microscopy lymphatic vessels and other tissues under the regulation of the Prox1 promoter (Fig. 2B). Using this technique, cervical lymph nodes that were anatomically consistent among all rats used for this study were identified and dissected as described in the “Lymphatic Injury Protocol” subsection.

### Lymphatic injury protocol

In order to create a novel head and neck lymphedema model, we applied similar principles used to create tail and limb lymphedema rodent models and performed lymphatic dissection with radiation therapy to produce subacute-to-chronic changes consistent with lymphedema development^21,22,26^. Under isoflurane-inhaled anesthesia, rats in the lymphadenectomy group received the lymphatic injury protocol, consisting of cervical lymph node removal followed by adjuvant radiation therapy (Fig. 2A). Using a dissecting microscope, a circumferential full-thickness skin incision was made across the neck to disrupt the dermal lymphatic network, followed by careful exploration of the soft tissue structures of the ventral neck to identify the mandibular lymph node (MLN), accessory mandibular lymph node (AMLN), and superficial parotid lymph node (SPLN) on each side (Fig. 2C,D). The deep cervical lymph node (DCLN) was exposed by reflecting the submandibular gland and dissected deep to the junction of the omohyoid (OH), sternohyoid (SH) and sternocleidomastoid (SCM) muscles (Fig. 2E,F). As previously described, sutures were placed in 7–8 mm gaps to reinforce discontinuity of dermal lymphatics. The surgical area was then irradiated using a previously established clinically relevant dose of 27.5 Gy delivered by an XRad 320 device over a period of 5 days (5.5 Gy per day) starting at post-operative day 1^23^.

### Post-operative measurements

Prior to surgery, all animals were age-and weight-matched to eliminate confounding variables. As previously described, at all time points animals were photographed using a Nikon D5000 digital SLR flash camera using fixed distance, focal length, background, and positioning^23^. Head and neck measurements, the primary outcomes to assess lymphedema development, were performed on the day of surgery (day 0) and post-operative days 15, 30, and 60. These included neck circumference and maximum face width. Neck circumference was measured with a tape measure, with guidance from bony landmarks. Maximum face width was measured as zygion-to-zygion distance using electronic calipers. To account for age-appropriate growth and differences in animal size, head and neck measures were normalized for each animal by calculating percent change relative to pre-operative measures using the formula (S_pre_ − S_post_)/S_post_ × 100, where S_pre_ is the pre-operative size and S_post_ is the post-operative size. All measurements were performed in triplicate and statistically analyzed as mean values.

### MRI fat volume analysis

Since early-stage HNL is associated with fatty deposition within the soft tissue structures of the head and neck, we chose to assess changes in fat content within the head and neck region between the two groups. Head and neck fat volume was calculated using T1-weighted magnetic resonance imaging (MRI) analysis. Briefly, under isoflurane-induced anesthesia, animals were immobilized and placed within an Aspect Imaging M7™ Mx Compact MRI machine (Aspect Imaging, Shoham, Israel). MRI scans of the head and neck were acquired using a 1 mm gap and analyzed using Amira software for 3D data visualization, processing and analysis (Thermo Fisher Scientific, Waltham, MA) to quantify total fat volume from the base of the ears to the sternum in mm^3^. To account for age-appropriate growth and differences in animal size, fat volume values were normalized for each animal by calculating percent change relative to pre-operative measures as previously described.

### Indocyanine green lymphography

On post-operative day 60, lymphatic clearance was measured using indocyanine green (ICG) lymphography to compare lymphatic function between the lymphadenectomy and sham groups. As we previously described, after inducing anesthesia via isoflurane inhalation, a sub-microliter injection system syringe was used to intradermally inject 2 µL of ICG at 3 different landmarks in the submandibular region proximal to the disrupted lymphatics (one submental injection and two injections below the angle of the mandible)^23^. ICG lymphography images were recorded using SPY-Q technology (Novadaq Technologies, BC, Canada) at various time points up to 144 hours post-injection. The fluorescence intensity of the ICG signal being drained from the ventral neck was quantified at each post-injection time point using ImageJ pixel intensity analysis (National Institutes of Health, Bethesda, MD).

### Histological assessment

As previously described, at euthanasia rat neck tissue was harvested and fixed in 10% neutral buffered formalin solution for 24 hours. The tissue was then decalcified using Surgipath Decalcifier II solution (Leica Biosystems Inc., Buffalo Grove, IL), embedded in paraffin, and sectioned using 3–5 μm tissue thickness. Sections were subsequently stained with hematoxylin and eosin (H&E) to document differences in histological architecture of the soft tissues of the neck between the two groups^23^. Dermal and subcutis thickness of the skin were measured with ImageJ using at least 3 images per cross-section taken at 20x magnification using a BZ-X800E microscope (Keyence Corporation of America, Itasca, IL). Sections were also stained with picrosirius red to detect differences in collagen deposition and fibrotic response between the two groups. Cross-sections were bathed in picrosirius red solution (Sigma-Aldrich, St. Louis, MO) for 40 minutes, followed by serial dehydration and mounting^23^. Collagen deposition was quantified by measuring the percentage of collagenous area (in red) out of the total dermal tissue area using at least 3 images per cross-section taken at 20x magnification.

### Immunohistochemical assessment

Rat neck samples that had been formalin-fixed and paraffin-embedded were immunostained for CD3, a pan T-lymphocyte cell-surface marker (catalog # ab5690; Abcam, Cambridge, MA), and for TGFβ1, a cell-surface marker and mediator of fibrosis (catalog # sc-130348; Santa Cruz Biotechnology, Inc., Dallas, TX) according to manufacturer recommendations. Briefly, sections were deparaffinized, heated in citrate buffer for antigen retrieval, and incubated overnight in primary antibody for CD3 (1:100) or TGFβ1 (1:100) at 4 °C in 5% bovine serum albumin blocking buffer. Afterward, sections were incubated in biotinylated secondary antibody (1:200) for 2 hours, then incubated with alkaline phosphatase-conjugated streptavidin and diaminobenzidine. In order to document differences in cell density, the number of CD3 positive cells or TGFβ1 positive cells was quantified on ImageJ using at least 3 images per cross-section taken at 40x magnification.

### Molecular assessment

Quantitative reverse transcription polymerase chain reaction (qRT-PCR) was performed to assess differential expression of TGFβ1 mRNA between rat neck tissue from lymphadenectomy and sham groups. Gene expression was normalized to glyceraldehyde-3-phosphate dehydrogenase (GADPH) mRNA levels as an endogenous control in qRT-PCR.

### Statistical analysis

For all outcomes, statistical analysis was conducted using GraphPad Prism 8.0 software (GraphPad Software, Inc., San Diego, CA) using a two-tailed Student’s *t*-test to compare the means of the lymphadenectomy and sham groups. As previously described, all outcomes were analyzed using mean values and standard error^23^. Statistical significance was determined by P-values less than 0.05.

## Results

### Combined cervical lymphatic injury results in cervical and facial expansion

**Figure 3:**
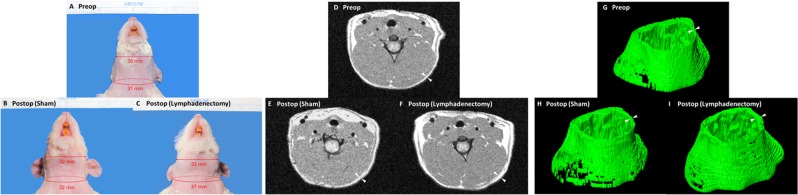
Representative pre- and post-operative images of the cervical lymphadenectomy and sham groups. (**A**) Representative pre-operative image taken at day 0. (**B**) Post-operative image of a sham rat taken at day 60. (**C**) Post-operative image of a lymphedenectomy rat taken at day 60. Note the increase in maximum face width and neck circumference compared to the pre-operative and post-operative sham rats. Note that the increase in maximum face width and neck circumference is greater post-operatively in the lymphadenectomy group than the sham group. (**D**) Representative pre-operative MRI scan acquired on day 0 demonstrating fat content within the head and neck region (white). (**E**) Post-operative MRI scan of a sham rat acquired on day 60. (**F**) Post-operative MRI scan of a lymphedenectomy rat acquired on day 60. White arrowheads indicate the subcutaneous fatty layer of the lateral neck. Note that the thickness of the subcutaneous layer is greater post-operatively in the lymphadenectomy group than the sham group. (**G**) Representative pre-operative 3D MRI reconstruction acquired on day 0 demonstrating fat content within the head and neck region (green). (H) Post-operative 3D MRI reconstruction of a sham rat acquired on day 60. (**I**) Post-operative 3D MRI reconstruction of a lymphedenectomy rat acquired on day 60. White arrowheads indicate the subcutaneous fatty layer of the lateral neck. Note that the thickness of the subcutaneous layer is greater post-operatively in the lymphadenectomy group than the sham group.

**Figure 4:**
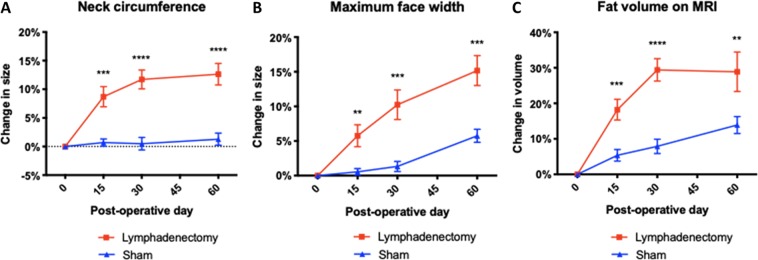
Lymphatic injury results in increased neck circumference, maximum face width, and fat volume on MRI. (**A**) On post-operative days 15, 30 and 60, the percent changes in neck circumference were 8% (P = 0.0002), 11% (P < 0.0001), and 11% (P < 0.0001) greater in lymphadenectomy animals than sham animals, respectively. (**B**) On post-operative days 15, 30 and 60, the percent changes in maximum face width were 5% (P = 0.003), 9% (P = 0.0004), and 9% (P = 0.0003) greater in lymphadenectomy animals than sham animals, respectively. (**C**) On post-operative days 15, 30 and 60, the percent changes in head and neck fat volume were 13% (P = 0.0006), 21% (P < 0.0001), and 15% (P = 0.009) greater in lymphadenectomy animals than sham animals, respectively.

After undergoing sham surgery or combined cervical lymphatic injury, control and lymphadenectomy rats, respectively, were observed until post-operative day 60. As shown in Fig. 3A–C, an increase in face width and neck size was visually appreciable in the lymphadenectomy group compared to the sham group by day 60. This was further corroborated by quantitative measurements taken every 15 days post-operatively of neck circumference and maximum face width. Since lymphedema in its early stages is known to result in adipose tissue hypertrophy, image analysis of MRI scans was performed to quantify the volume of fat present within the head and neck region of lymphadenectomy and sham rats. As shown in Fig. 3D–I, an increase in fat content within the subcutaneous and deeper soft tissue structures of the head and neck was appreciable in the lymphadenectomy group compared to the sham group by day 60. Changes in head and neck measurements were analyzed within each group by comparing each postsurgical time point to day 0, allowing us to assess the degree of head and neck expansion over time between the two groups (Fig. 4A–C). The control group demonstrated baseline increase of head and neck measurements over the 60 day period as evidenced by an increase in the percent change over time for all three measures (blue line). However, the lymphadenectomy group demonstrated significantly more expansion across all measures when compared to the sham group by day 60 (red line). Statistical analysis revealed significant increases in neck circumference, maximum face width and fat volume over time in lymphadenectomy animals beginning on post-operative day 15 and lasting until the final time point. Compared to the sham group, by post-operative day 60 neck circumference was 11% greater (P < 0.0001), maximum face width was 9% greater (P = 0.0003), and fat volume was 17% greater (P = 0.04) in the lymphadenectomy group. In summary, animals that underwent the combined cervical lymphatic injury protocol experienced post-operative head and neck swelling and fat deposition consistent with lymphedema.

### Delayed lymphatic clearance in the head and neck after cervical lymphadenectomy

**Figure 5:**
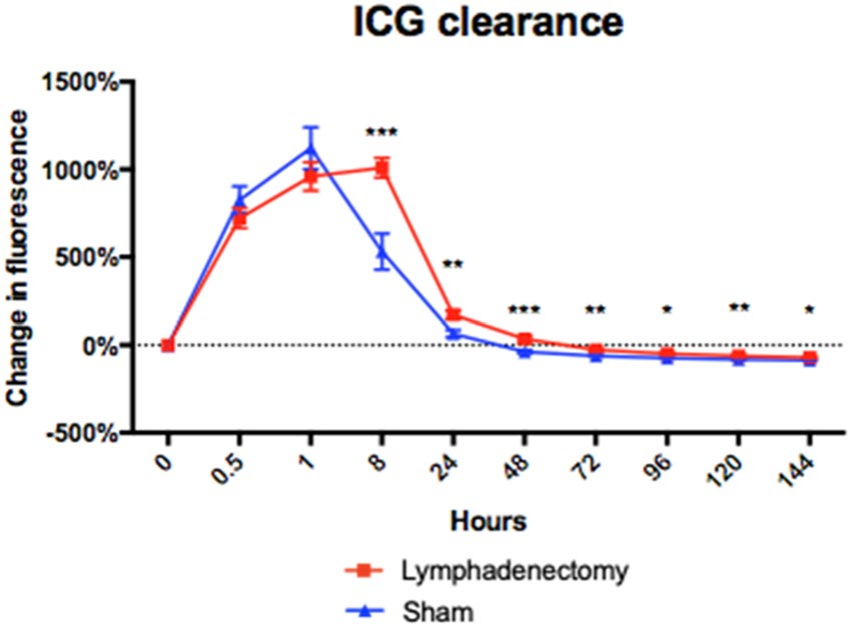
Lymphatic injury results in delayed lymphatic clearance. ICG lymphography of lymphadenectomy and sham animals. Change in fluorescence from injection time 0 plotted as a function of time (in arbitrary units). Compared to sham animals, lymphadenectomy animals demonstrate significantly faster lymphatic drainage at 8, 24, 48, 72, 96, 120 and 144 hours following ICG injection (P < 0.05).

Indocyanine green (ICG) lymphography is a technique widely used in clinical practice that allows for real-time visualization of lymphatic vessels for diagnostic or pre-operative planning purposes. It has been a useful tool for the evaluation of secondary lymphedema with sensitivity and specificity nearing 100%^27^. When injected intradermally, ICG is preferentially taken up by the lymphatic system; thus, lymphatic clearance can be visualized and measured using ICG fluorescent signal change over time. We hypothesized that animals that had undergone the combined cervical lymphatic injury protocol would experience delayed lymphatic clearance compared to animals that underwent sham surgery. At post-operative day 60, ICG lymphographic analysis was performed by quantifying the change in dermal fluorescence of the rat neck region injected with ICG at time 0. ICG clearance data revealed significantly delayed lymphatic clearance in the lymphadenectomy group compared to the control group (Fig. 5). After reaching peak fluorescence intensity at 1 hour post-injection, the lymphatics of the sham group began to drain ICG and lose signal intensity from hour 8 to hour 144. In contrast, at hour 8 post-injection, the lymphadenectomy group exhibited a persistence of fluorescence signal within the neck region injected with ICG. Subsequently, the lymphadenectomy group retained significantly more fluorescent signal until hour 144 compared to the sham group, indicating impaired lymphatic clearance of ICG. Thus, the cervical lymphatic injury protocol results in persistently decreased rates of lymphatic clearance, which is consistent with the development of head and neck lymphedema.

### Combined cervical lymphatic injury protocol results in subcutaneous tissue swelling, chronic inflammation, and fibrosis characteristic of lymphedema

**Figure 6:**
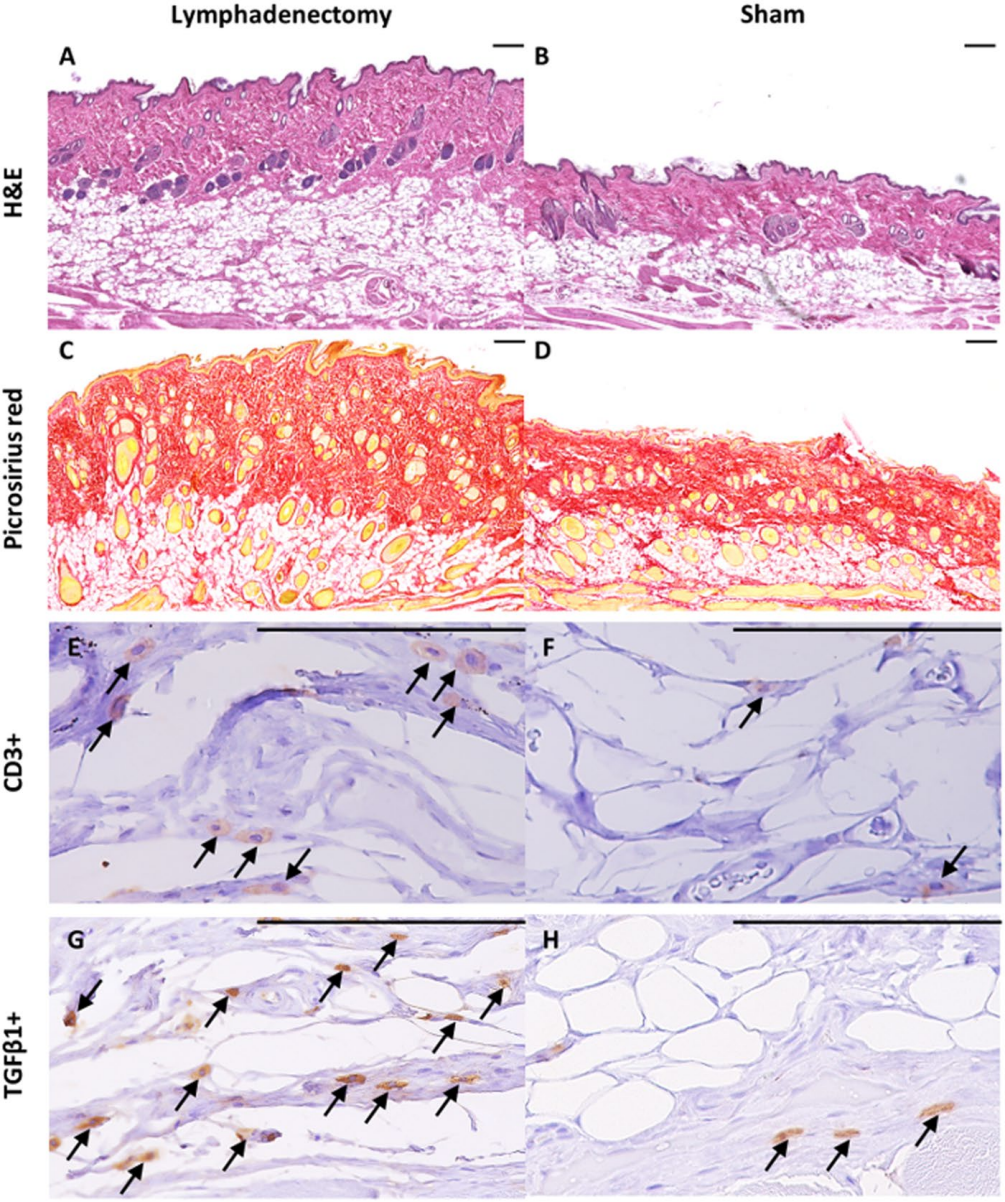
Lymphatic injury results in characteristic subcutaneous tissue expansion, collagen deposition, and T-cell infiltration as seen in human lymphedema. (**A**) Representative H&E stained cross-section of rat neck skin from the lymphadenectomy group. Horizontal scale bars represent 100 μm. (**B**) Representative H&E stained cross-section of rat neck skin from the sham group. Note the increase in dermal and subcutis thickness in lymphadenectomy animals compared to sham animals. (**C**) Representative picrosirius red stained cross-section of rat neck skin from the lymphadenectomy group. Horizontal scale bars represent 100 μm. (**D**) Representative picrosirius red stained cross-section of rat neck skin from the sham group. Note the increase in collagen deposition in lymphadenectomy animals compared to sham animals. (**E**) Representative CD3 stained cross-section of rat neck skin from the lymphadenectomy group. Horizontal scale bars represent 100 μm, CD3+ T-lymphocytes can be appreciated in brown. (**F**) Representative CD3 stained cross-section of rat neck skin from the sham group. Note the increase in CD3+ lymphocytic infiltration in lymphadenectomy animals compared to sham animals.

**Figure 7:**
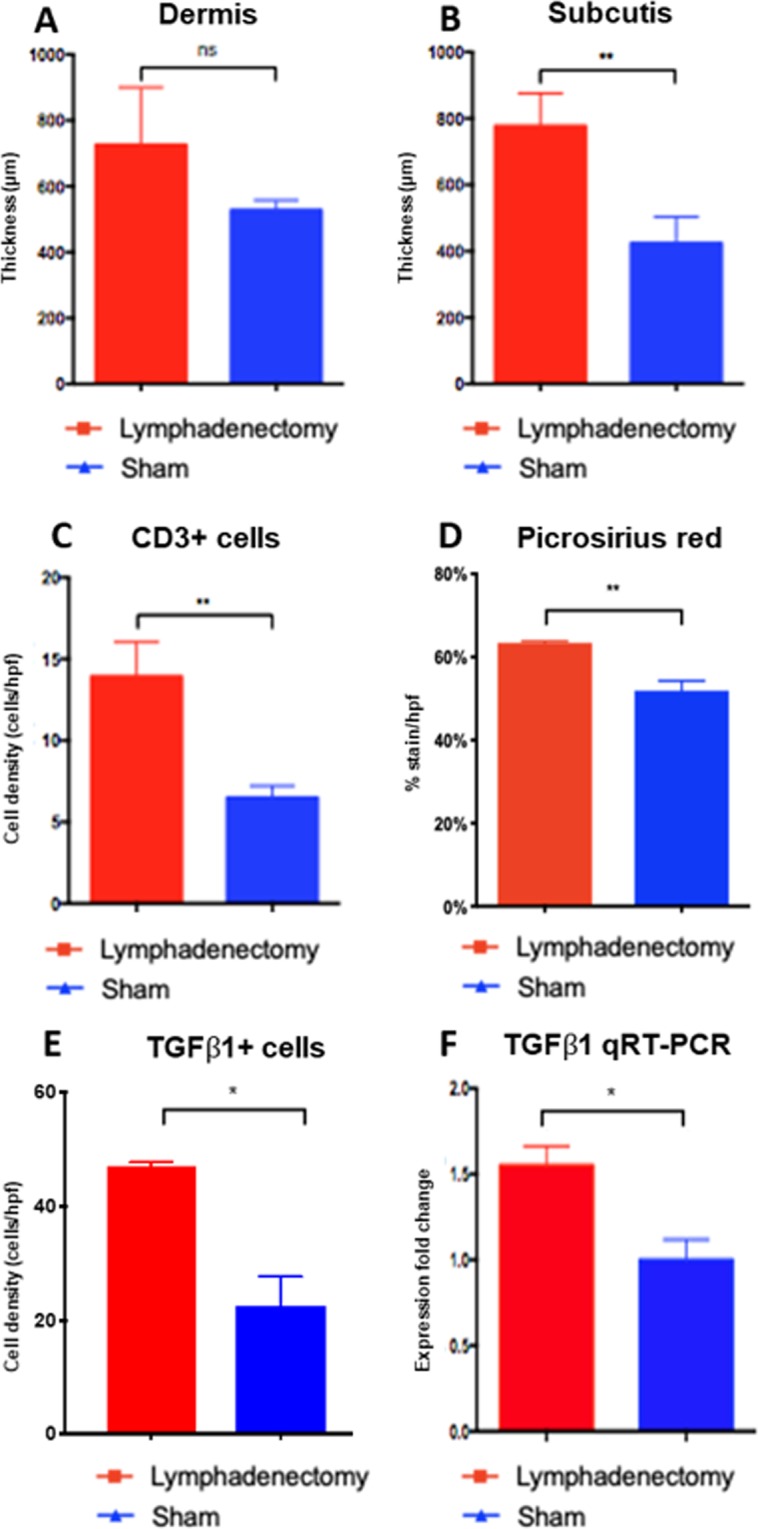
Lymphatic injury results in characteristic fatty deposition, inflammation and fibrosis as seen in human lymphedema. (**A**) H&E analysis of neck tissue from lymphadenectomy rats reveals a 38% increase in dermis thickness compared to sham rats (P = 0.12) (**B**) H&E analysis of neck tissue from lymphadenectomy rats reveals an 83% increase in subcutis thickness compared to sham rats (P = 0.008) (**C**) Quantified IHC sections of CD3-stained tissues reveal a 114% greater CD+ T-lymphocyte density in the lymphadenectomy group compared to controls (P = 0.005). (**D**) Picrosirius red staining of neck tissue from lymphadenectomy rats reveals a 22% increase in collagen density per total tissue area compared to sham rats (P = 0.001). (**E**) Quantified IHC sections of TGFβ1-stained tissues reveal a 110% greater TGFβ1 +cell density in the lymphadenectomy group compared to controls (P = 0.04). (**F**) Quantified reverse transcriptase polymerase chain reaction of rat neck tissue reveals a 1.7-fold change in TGFβ1 mRNA expression in the lymphadenectomy group compared to controls (P = 0.03).

Lymphedema is characterized by (1) expansion of subcutaneous tissue via fluid and fat deposition, (2) chronic inflammation, and (3) tissue fibrosis. To measure each of these parameters, we conducted histological and immunohistochemical analysis of neck tissue in the sham and lymphadenectomy animals at day 60. Dermal and subcutaneous tissue swelling was quantified by measuring the thickness of the dermis and subcutis in H&E sections of sham and lymphadenectomy animal neck tissue (Fig. 6A,B). Animals that underwent lymphadenectomy demonstrated 38% greater dermal thickness (P = 0.12) and 83% greater subcutis thickness (P = 0.008) compared to controls (Fig. 7A,B). Antibody staining for CD3, a pan T-lymphocyte marker, was used to quantify T-lymphocytic infiltration of the neck skin and thus to assess the degree of inflammation, which is known to be central to the pathogenic mechanisms of lymphedema (Fig. 6E,F)^28–30^. Indeed, lymphadenectomy animals had 114% greater levels of T-cells within the dermis compared to controls (P = 0.004), indicating a larger degree of inflammatory infiltration (Fig. 7C). Lastly, tissue fibrosis was quantified by measuring collagen deposition in the neck skin via picrosirius red staining (Fig. 6C,D) and antibody staining for TGFβ1, a cellular marker and mediator of fibrosis. Animals that underwent lymphadenectomy demonstrated 22% greater picrosirius red staining (P = 0.001) (Fig. 7D) and 110% greater TGFβ1 + cell density compared to controls (P = 0.04) (Fig. 7E). The rise in TGFβ1 protein expression in the lymphedenectomy group was further confirmed with qRT-PCR analysis revealing a 1.7-fold change in TGFβ1 mRNA expression compared to controls (P = 0.03) (Fig. 7F). The significant increase in subcutis thickness, T-cell density, collagen deposition, and TGFβ1 expression suggests that the combined cervical lymphatic injury protocol mediates chronic changes that are consistent with postsurgical lymphedema seen in humans.

## Discussion

Head and neck lymphedema (HNL) is a common complication of head and neck cancer treatment, affecting over 90% of patients^7,31^. Lymphatic disruption in HNL due to the cancer itself, tumor resection, or lymphadenectomy can result in chronic swelling of the external and/or internal soft tissue structures in the head and neck. Symptoms range from impaired speech, swallowing, and breathing to pain and body image issues^10,14^. Despite its high prevalence among head and neck cancer patients, HNL remains a challenging disease to diagnose and manage. Unlike lymphedema of the extremities, lymphedema in the head and neck is less likely to receive early diagnosis and timely treatment due to limited awareness on the part of clinicians and patients, as well as variability in clinical manifestation. As a result, patients are at risk of being underdiagnosed or diagnosed in the late stages of the disease, when tissues can become fibrotic and result in severe disfigurement and resistance to conservative management^32^. Currently, there is no standardized treatment regimen specific to HNL. Recently, the use of surgical and pharmacological treatments for lymphedema has garnered scientific interest given their potential for prophylaxis or mitigating the development of severe disease. However, data on animal studies or clinical trials for surgical and pharmacological treatments specific to HNL is lacking in the literature^32^. While most translational research in the field of lymphedema is done on limb and tail lymphedema animal models, there has not been a reliable, cost-efficient, and reproducible animal model of HNL. Without an animal model to reproduce HNL, the etiology and disease progression have not been well established in the scientific literature, thus preventing advancements in treatment for this debilitating disease. Overall, a lack of fundamental understanding of HNL in both the medical and research communities has had a negative impact on the development of effective treatment modalities for this condition.

In this study, we demonstrate what we believe to be the first reproducible animal model for HNL. On gross examination, animals that received the combined cervical lymphatic injury protocol showed a significant degree of head and neck swelling consistent with lymphedema progression. The head and neck MRI scans of lymphadenectomy animals also showed a significant increase in fat deposition over time, a hallmark of lymph stasis and lymphedema progression^33^. The cervical lymphatic injury protocol resulted in delayed lymphatic clearance on ICG lymphography, which is consistent with HNL development. Histological findings in lymphadenectomy animals were further consistent with postsurgical lymphedema seen in humans. According to the most recent consensus document by the International Society of Lymphology, deposition of fat and fibrotic tissue becomes more evident with lymphedema progression^33^. In previous animal models, it has been shown that chronic inflammation mediated by the Th2 differentiation pathway plays a significant role in lymph stasis and fibrosis in lymphedema^30^. The significantly increased subcutis thickness observed in lymphadenectomy animals is consistent with the increased MRI fat volume observed at day 60, suggesting expansion of the subcutaneous tissue due to increased fat deposition caused by lymphedema. Moreover, the significantly increased collagen deposition in the lymphadenectomy group is consistent with an increased fibrotic response, as confirmed by elevated TGFβ1 mRNA and protein expression. Lymphadenectomy animals also demonstrated significantly increased markers of inflammation, as evidenced by greater CD3 positive T-cell staining. Thus, histological findings of increased fat deposition, fibrosis and inflammation in animals that received the combined cervical lymphatic injury protocol further suggest that the swelling seen on gross exam indicates postsurgical lymphedema.

Although it has a high prevalence within the head and neck cancer community, HNL is associated with relatively limited awareness and research efforts. As such, we believe that the model described in this study serves as a first step into the exploration of discovering curative therapies for this disease. Alternative therapies for lymphedema, such as surgical procedures that restore lymph flow and pharmacologic agents that promote lymphangiogenesis, are currently being studied^34–39^. Research on potential pharmacologic agents such as vascular endothelial growth factor C (VEGF-C), stem cells, and 9-*cis* retinoic acid (alitretinoin), show great promise in lymphedema reduction in other animal models of the disease. VEGF-C and VEGF-D are lymphatic-specific growth factors that have demonstrated favorable properties in reducing lymphedema development in small and large animal models^40–46^. While there have been extensive small and large animal studies on the therapeutic potential of VEGF-C, some studies have also reported its link to tumor metastasis. This may limit the clinical utility of VEGF-C in post-oncologic patients, who represent a significant proportion of lymphedema patients^47,48^. Nine-*cis* retinoic acid (9-*cis* RA or alitretinoin) is an alternative pharmacologic agent that has been shown to increase lymphatic clearance and lymphangiogenesis in mouse tail and hind limb models^49,50^. Because it is already approved for clinical use by the US Food and Drug Administration (FDA), 9-*cis* RA has potential to be repurposed for prophylactic use in postsurgical extremity lymphedema in patients.

Currently, there is a lack of research focus on identifying preventative or curative agents for the progression of head and neck lymphedema. While much of the current translational research on potential pharmacologic agents for lymphedema seems promising, all of these studies have been conducted using models of lymphedema not specific to the head and neck region, such as mouse tail models and small and large animal hind limb models. While an animal model for head and neck lymphedema may be helpful to test such pharmacologic agents, one limitation is that it may not allow for the testing of conservative treatments, such as manual compression devices, due to the inherent differences in size and contour of rat necks compared to human necks. Testing pharmacologic agents that affect wound healing using the described model also has the potential to confound results if the agents are administered concurrently with radiation, as they might alter the cellular and physiologic effects of radiation injury. Nevertheless, the animal model described in this study not only accurately recapitulates the clinical, physiologic, and histopathologic features of human HNL, but is also easily and highly reproducible. Of all of the rats that received the combined cervical lymphatic injury protocol, 100% developed lymphedema as seen on gross exam and histology.

Lastly, it is important to note that many head and neck patients develop HNL as a result of tumor resection or radical lymph node dissection that disrupts the lymphatic circulation only unilaterally or at select anatomical neck levels (superficial or deep lymphatic networks). Our work provides the first step toward establishing a reproducible animal model of HNL that develops from a more aggressive extent of lymphatic injury and, thus, exhibits more severe symptoms. Nevertheless, our group has already begun investigating future HNL animal models that could produce more clinically relevant symptoms with less aggressive lymphatic disruption. Moreover, while we induced post-operative radiation in our animals to simulate post-operative radiation in head and neck cancer patients, not all head and neck patients receive radiation. Thus, it is still unknown whether the resection of only specific lymph nodes unilaterally and in select anatomical levels is sufficient to produce results, or whether HNL can develop without injury caused by post-operative radiation. Future work is still needed to finetune lymphatic injury protocols that have the potential to provide more clinically relevant symptoms. Ultimately, our novel animal model of HNL serves a foundation upon which this disease can be studied in greater depth as well as a platform to evaluate future therapeutics specific to head and neck lymphedema.

## Data Availability

The data that support the findings of the studies referenced in this article are openly available in PubMed and/or PubMed Central.
